# Multi-AGV path planning with double-path constraints by using an improved genetic algorithm

**DOI:** 10.1371/journal.pone.0181747

**Published:** 2017-07-26

**Authors:** Zengliang Han, Dongqing Wang, Feng Liu, Zhiyong Zhao

**Affiliations:** 1 College of Automation and Electrical Engineering, Qingdao University, Qingdao, 266071, P.R. China; 2 Department of Industrial Engineering, University of Texas at Arlington, Arlington, TX 76019, United States of America; Chongqing University, CHINA

## Abstract

This paper investigates an improved genetic algorithm on multiple automated guided vehicle (multi-AGV) path planning. The innovations embody in two aspects. First, three-exchange crossover heuristic operators are used to produce more optimal offsprings for getting more information than with the traditional two-exchange crossover heuristic operators in the improved genetic algorithm. Second, double-path constraints of both minimizing the total path distance of all AGVs and minimizing single path distances of each AGV are exerted, gaining the optimal shortest total path distance. The simulation results show that the total path distance of all AGVs and the longest single AGV path distance are shortened by using the improved genetic algorithm.

## Introduction

Multi-objective optimization have been applied in many fields, including engineering [1–5], transportation [6–10] and logistics [11–15]. A multi-objective problem is to search for a solution that satisfies all objective functions between conflicting objectives. Multi-objective optimization methods include classical optimization algorithms (like weighted sum methods, *ε*-constraint methods, interactive methods, Pareto-dominated methods) [16–19] and intelligent optimization methods (like evolution based algorithms and swarm based algorithms) [20–24].

Multiple automated guided vehicles (multi-AGVs), characterized by multi-objectives, are playing an increasingly important role in the area of distribution logistics due to their high efficiency for material handling among workstations. The applications of AGV systems face several important issues: AGVs number determining [25–27], path planning [28–30] and constraint exerting [31–33], etc.

Determining the optimal numbers of vehicles is the fundamental problem in the management of an AGV system. Several methodologies have been proposed to achieve this goal and their main objective is to attend all tasks on time with a sufficient numbers of vehicles [25–27, 34]. For example, Vivaldini et al. presented a new module to estimate the optimal numbers of AGVs for the execution of a set of tasks by integrating task assignment and routing [27]. Ji and Xia built a new model for an AGV system and studied the minimum vehicle numbers by an approximately analytical method based on the binary search [34]. Koo et al. studied an AGV fleet size model where part waiting time is estimated for various vehicle dispatching rules to determine the proper AGV fleet size [26].

The multi-AGV path planning is most important in ensuring an efficient flow of materials during the production process. The path planning involves three issues in dispatching, scheduling and routing of tasks at the same time. The multi-AGV path planning problem [35–37] is similar to the traveling salesman problem (TSP) [38–40] in the aspect of finding the shortest tour/time which has extremely large search spaces and is very difficult to solve. Smolic-Rocak et al. used time windows in a vector form to solve the shortest path problem for multi-AGV systems [36]. Draganjac et al. implemented a shortest feasible path planning algorithm considering nonholonomic vehicle constraints for multi-AGV systems [35]. Wang et al. proposed a multi-offspring genetic algorithm for the TSP by producing excellent individuals [38]. Wang et al. investigated a novel memetic algorithm with a competitive capacity to maintain the total distance as short as possible for the TSP [39]. Jiang and Yan developed a discrete fruit fly optimization algorithm for the TSP [40].

There existing various constraints in multi-AGV path planning, e.g. collision-free constraints [41–43], time window constraints [36, 44], and time/distance constraints [35, 45]. This paper investigates an improve genetic algorithm for multi-AGV path planning by exerting double-path restrictions on both the total path distance of all AGVs and single path distances of each AGV, and by choosing three-exchange crossover heuristic operators for crossover operation.

Traditional genetic algorithms adopted two-exchange crossover operators for crossover operation, that is, using two parent individuals to produce a progeny chromosome [46–48]. Obviously, traditional genetic algorithms with two parent individuals other than more parent individuals would obtain less parent information, and reduce the diversity of offspring performance. In order to improve the diversity of progenies, we put forward the idea of three-exchange crossover operators for crossover operation, that is, using three parent individuals to produce a progeny chromosome.

The contributions of this paper lie in the follows:

- Unlike the traditional path constraint only exerting on the total path distance of all AGVs, this paper exerts double-path restrictions on both the total path distance of all AGVs and single path distances of each AGV. The strategy double shortened total/single AGV path distance and obtained the optimal result.
- By using three parent individuals other than the traditional method with two parent individuals, to produce a progeny chromosome, this method increases the information of producing the progeny chromosomes, earning the possibility of inheriting the excellent characteristics of the parents and accelerating the searching speed of the improved algorithm.
- Simulations on multi-AGV path diagrams and the iterative maps of the improved/traditional genetic algorithms verify that the improved genetic algorithm has the shorter total path distance of all AGVs than that of the traditional genetic algorithm.

The rest of the paper is organized as follows. Section 2 demonstrates the overview of facility layout. Section 3 investigates an improved genetic algorithm with double-path constraints by using the three-exchange crossover heuristic operators. Section 4 provides the feasibility of the algorithm by simulation. Finally, the concluding remarks are involved in Section 5.

## Overview of facility layout

### Facility layout

A jobshop manufacturing system with multiple AGVs performs material delivering. There are *M* AGVs traversing through *N* workstations (*N* > *M*). For the workstation distribution, the following assumptions are considered for describing the details.

- *M* AGVs traverse through *N* workstations (*N* > *M*).
- Only one AGV passes through each workstation (except the starting point).
- Each AGV starts from the same starting point (workstation) and comes back to the starting point.
- Each AGV travels one route separately with the predefined path and the fixed speed.
- Two constraints are exerted:
  The total path distance of all AGVs should be minimized;
  Each single AGV path distance should be minimized.

**Fig 1:**
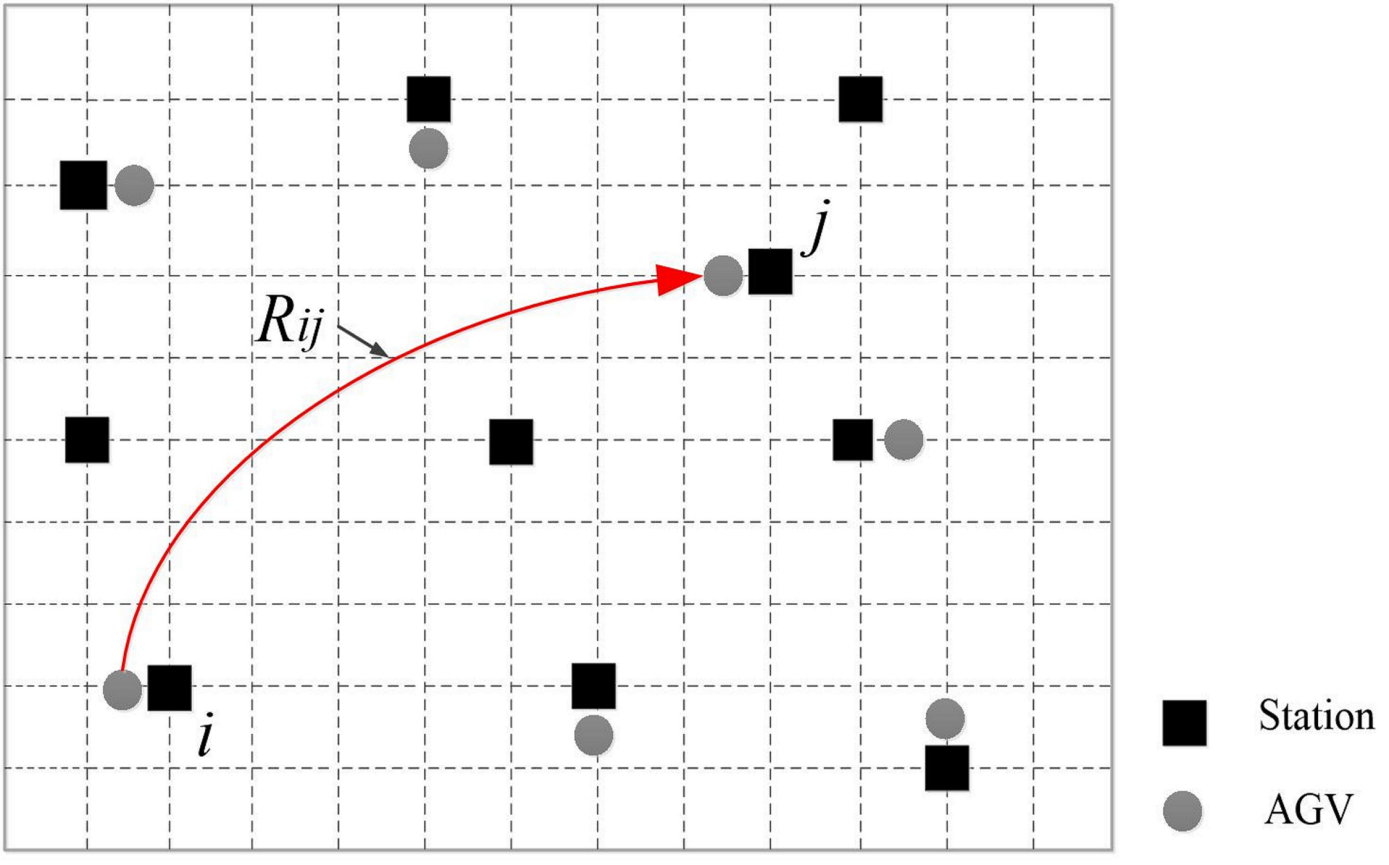
The work diagram of the AGV system.

The schematic diagram is shown in Fig 1.

### Mathematical formulation

Referring to Fig 1, we formulate the proposed problem, mathematically. The indexes, parameters and variables are introduced below.

1. Indexes
  *i*, *j*—Indexes for two workstations on the ends of an arc, *i* = 0, 1, 2 …, *N*, *j* = 0, 1, 2 …, *N*.
  *k*—Index for AGVs numbers, *k* = 1, 2 …, *M*.
  *l*—Index for the workstations requires AGVs to delivery, *l* = 1, 2 …, *L*_*k*_, 0 < *L*_*k*_ < *N*.
2. Parameters
  *R*_*ij*_—Arc between two workstations *i* and *j*.
  *C*_*ij*_—Path through the corresponding arc segment *R*_*ij*_.
3. Variables
  For *i* = 0, 1, …, *N*, *j* = 0, 1, …, *N*, *k* = 1, …, *M*, define
  $$\begin{array}{c} X_{ijk}=\left\{ \begin{array}{cc} 1 & \text{the}\;\text{kth}\;\text{AGV}\;\text{passes}\;\text{the}\;\text{arc}\;R_{ij}\\ 0 & \text{otherwise}, \end{array}\right. \end{array}$$ (1)
  $$\begin{array}{c} Y_{ki}=\left\{ \begin{array}{cc} 1 & \text{the}\;\text{kth}\;\text{AGV}\;\text{goes}\;\text{to}\;\text{the}\;\text{ith}\;\text{station}\\ 0 & \text{otherwise}, \end{array}\right. \end{array}$$ (2)
  $$\begin{array}{c} z_{k}=\sum_{i=0}^{L_{k}}\sum_{j=0}^{L_{k}}C_{ij}X_{ijk}. \end{array}$$ (3)
4. Objective function
  $$\begin{array}{c} J=min\left(\sum_{k=1}^{M}z_{k}\right)\cap minz_{k}\;\left(k=1,2,...,k\right), \end{array}$$ (4)
5. Requirements
  $$\begin{array}{c} \sum_{k=1}^{M}Y_{ki}=\left\{ \begin{array}{cc} M, & i=0\\ 1, & i=1,...,L_{k}, \end{array}\right. \end{array}$$ (5)
  $$\begin{array}{c} \sum_{i=0}^{L_{k}}X_{ijk}=Y_{kj},\;j=0,...,L_{k},\;k=1,...,M, \end{array}$$ (6)
  $$\begin{array}{c} \sum_{j=0}^{L_{k}}X_{ijk}=Y_{ki},\;i=0,...,L_{k},\;k=1,...,M, \end{array}$$ (7)

where

Requirement (5) specifies that each AGV starts from the starting workstation 0, all workstations can only be accessed once by an AGV;

Requirement (6) shows that any arc starts from the starting workstation;

Requirement (7) implies that any arc ends with the starting workstation.

## Algorithm design

The key problem of applying the genetic algorithm to the multi-AGV path planning is to adopt the effective coding and decoding methods. Genetic algorithms repeatedly select, crossover, and mutate the population to produce a new generation population that is more adaptable to the environment than its parents, until satisfying the desired requirements.

The step of the genetic algorithm includes: genetic coding, population selection, fitness function, selection action, crossover operation, and matrix decoding. The proposed new genetic algorithm minimizes the total path distance of all AGVs by selecting individuals with big fitness values, and minimizes each AGV path distances by the three-exchange heuristic crossover operator method.

### Genetic coding

Symbol 0 indicates the starting workstation (point); symbols 1, 2, …, *N* mean the *N* workstations that need AGVs delivery. We add *M* − 1 dummy symbols, denoting *M* − 1 virtual sites, labeled *N* + 1, …, *N* + *M* − 1. They have the same coordinates as the starting site, meaning that every time a dummy symbol appears, the corresponding AGV returns to the starting point. Assume that a gene represents a path that an AGV travels; one chromosome contains all genes, i.e., all paths that all AGVs travel. To avoid frequent sub-paths, we assume that the path distance from the starting point 0 to the starting point 0 is infinite.

**Fig 2:**
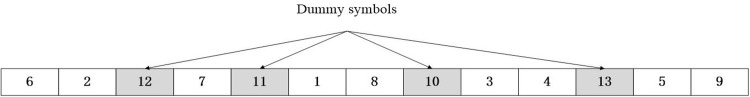
A random chromosome.

For example: there are 10 workstations, the code is 0-9, 5 AGVs to complete the task, a random chromosome coding is shown in Fig 2.

The paths of the five AGVs are as follows:

0 – −6 – −2 – −0

0 – −7 – −0

0 – −1 – −8 – −0

0 – −3 – −4 – −0

0 – −5 – −9 – −0

**Fig 3:**
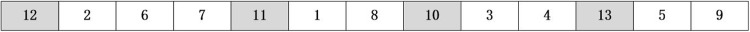
Situation 1 of dead solutions.

**Fig 4:**
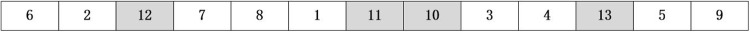
Situation 2 of dead solutions.

In the iterative process, there may be two kinds of dead solutions as the following Figs 3 and 4.

1. The virtual symbols are on one end of a chromosome
  The paths of the five AGVs are as follows:
  0 – −0 – −0
  0 – −2 – −6 – −7 – −0
  0 – −1 – −8 – −0
  0 – −3 – −4 – −0
  0 – −5 – −9 – −0
  The 0 – −0 – −0 path means the distance is infinite, and can not meet the distance minimizing constraint, so this chromosome will be eliminated.
2. The virtual symbol appears continuously in a chromosome
  The paths of the five AGVs are as follows:
  0 – −6 – −2 – −0
  0 – −7 – −8 – −1 – −0
  0 – −0 – −0
  0 – −3 – −4 – −0
  0 – −5 – −9 – −0
  Obviously, the 0 – −0 – −0 path is present on this chromosome, and can not meet the distance minimizing constraint, so this chromosome also will be eliminated.

### Population selection

The appropriate population size is important for the convergence of the genetic algorithm. If the population size is too small, the genetic algorithm is easy to converge to the local optimal solution; on the contrary, if the population scale is too large, the computing speed of the genetic algorithm will be reduced. The size of the population is related to the variable *N*, and the appropriate population size should be controlled between 4*N* and 6*N* [49].

### Fitness function

In this paper, we use the exponential fitness functions according to [50]. The idea of this transformation method comes from the SA (simulated annealing) process [50]. Due to the advantages of exponentiation scale transformation, referring to [51], we choose a fitness function with exponentially transformation as:

$$\begin{array}{c} f=\alpha *\exp(\beta *Z), \end{array}$$ (8)

where *Z*(= *Z*_1_+*Z*_2_+⋯+*Z*_*k*_) is one of the parent individuals; *α* and *β* are arithmetic constants; *α* determines the coercion of replication, the smaller the value, the greater the replication intensity of the individual with the greatest fitness.

### Selection operation

The selection operation is used to determine the recombination or crossover parent individuals and the number of offspring individuals generating by the candidate population. How to select an operator will directly affect the results of the genetic algorithm. An inappropriate operator will cause the evolution to stop or make the algorithm lose diversity, and produce premature problems [52].

In this paper, we use the Roulette Wheel Selection [53] to select the parent individuals, the probability of individual *i* is equal to the proportion of its fitness value and the sum of the individual population fitness [54], as the following,

$$\begin{array}{c} P_{i}=f_{i}/\sum_{k=1}^{Q}f_{k}, \end{array}$$ (9)

where *f*_*i*_ is the fitness value of the individual *i*. *Q* is the numbers of selected chromosomes or population size.

### Crossover operation

Traditionally, crossover refers to the process in which two chromosomes exchange some genes with each other in a certain way to form one new individuals. After crossover operation, a new generation is produced, and it inherits the father’s basic characteristics.

**Fig 5:**
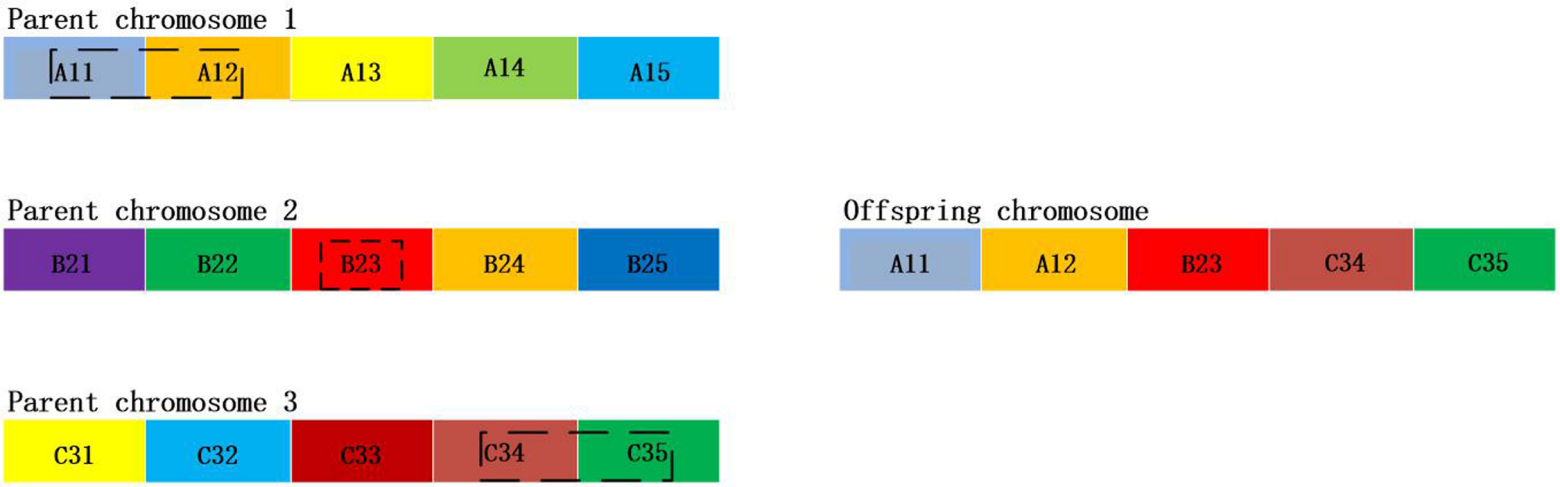
Three-exchange heuristic crossover operator method with 5 genes.

The idea of the three-exchange heuristic crossover operator method is to produce a progeny with three parent individuals. The proposed method increases the information of producing the progeny chromosomes, comparing with the traditional method with two parent individuals. The increased parent chromosomes improve the possibility of inheriting the excellent characteristics of the parents, and accelerate the searching speed of the algorithm. The explanation of the three-exchange heuristic crossover operator method is shown in Fig 5.

Taking a task including 10 workstations and 5 AGVs as an example, the process of three-crossover heuristic crossover operator method is described in detail as follows. The distance between the ten workstations is shown in Table 1.

**Table 1:** The distance between the ten workstations.

**start**	0	1	2	3	4	5	6	7	8	9
0	∞	4	1	12	7	5	6	3	5	5
1	5	0	7	4	1	8	1	2	4	7
2	3	5	0	3	1	6	4	9	1	3
3	7	1	9	0	7	8	9	5	9	9
4	8	6	6	1	0	13	5	1	3	12
5	1	4	7	3	2	0	5	2	5	2
6	9	7	8	8	7	1	0	1	11	4
7	12	11	3	2	7	1	6	0	3	8
8	5	4	2	4	4	5	3	5	0	7
9	2	7	5	11	8	7	9	8	4	0

Note: 0 is the starting point.

Three individuals were randomly selected as three-crossover heuristic crossover operators:

*A* = 6 2 12 7 11 1 8 10 3 4 13 5 9

*B* = 3 2 12 7 9 11 1 4 13 5 10 6 8

*C* = 5 3 10 8 7 11 2 6 12 1 4 13 9

The total distance of route *A* is 81, the largest distance of the single AGV path is 27;

The total distance of route *B* is 78, the largest distance of the single AGV path is 24;

The total distance of route *C* is 71, the largest distance of the single AGV path is 22.

Taking chromosome *A* as a reference, point 6 is the first position of chromosome *A*. From right to left, cyclically move genes in the chromosomes *B* and *C*, and stop when point 6 being the first position, then choose 6 as the first point of progeny *S*, the results are

*A* = 6 2 12 7 11 1 8 10 3 4 13 5 9

*B* = 6 8 3 2 12 7 9 11 1 4 13 5 10

*C* = 6 12 1 4 13 9 5 3 10 8 7 11 2

*S* = 6 ∗ ∗ ∗ ∗ ∗ ∗ ∗ ∗ ∗ ∗ ∗ ∗

From the Table 1, we can get the distance around point 6 as follows,

$$\begin{array}{c} d(6,2)=8, \end{array}$$

$$\begin{array}{c} d(6,8)=11, \end{array}$$

$$\begin{array}{c} d(6,12)=d(6,0)=9. \end{array}$$

Thus we get

$$\begin{array}{c} d(6,8)>d(6,12)>d(6,2). \end{array}$$

To meet the constraint of minimizing single AGV path distance, we choose point 2 as the second point, the results are

*A* = 6 2 12 7 11 1 8 10 3 4 13 5 9

*B* = 6 2 12 7 9 11 1 4 13 5 10 8 3

*C* = 6 2 12 1 4 13 9 5 3 10 8 7 11

Similarly, we can determine the other genes of crossover progeny *S* in turn. Exerting with the single AGV path distance minimizing constraint, the crossover progeny *S* of the first crossover step is

*S* = 6 2 12 7 9 11 1 4 13 5 10 8 3

The above obtained progeny chromosome indicates that the total path distance of all AGVs is 65 and the maximum path distance of a single AGV is 17. Obviously, the total path distance of all AGVs and the maximum single AGV path distance of the obtained *S* are less than those of original *A*, *B*, and *C*.

### Mutation operation

Mutation is to exchange genes within the same chromosome, resulting in a new individual. Mutation can determine the local search ability of the genetic algorithm, maintain the diversity of the group, prevent premature convergence of the genetic algorithm [55].

This paper adopts the exchanging mutation method [56]. The idea is to choose the ordinal numbers *a*, *b*, *c* (*a* < *b* < *c*), and then insert the intermediate paths *a* and *b* (including *a* *b*) after *c* (*c* refers to the paths associated with it). For example, if the sequence numbers *a* = 2, *b* = 5 and *c* = 10 are randomly generated, the corresponding individual transformations are as follows:

*A* = 6 2 12 7 11 1 8 10 3 4 13 5 9

After the mutation:

*A* = 6 1 8 10 3 4 2 12 7 11 13 5 9

### Matrix decoding

**Fig 6:**
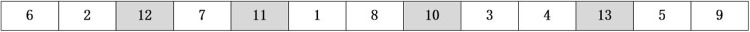
A random chromosome.

Set up a production scene with 10 workstations and 5 AGVs. A random chromosome is shown in Fig 6.

The paths of the five AGVs are:

0 – −6 – −2 – −0

0 – −7 – −0

0 – −1 – −8 – −0

0 – −3 – −4 – −0

0 – −5 – −9 – −0

According to Table 1, establish a 10 ∗ 10 workstation matrix *D*:

$$\begin{array}{c} D=(\begin{array}{cccccccccc} \infty & 4 & 1 & 12 & 7 & 5 & 6 & 3 & 5 & 5\\ 5 & 0 & 7 & 4 & 1 & 8 & 1 & 2 & 4 & 7\\ 3 & 5 & 0 & 3 & 1 & 6 & 4 & 9 & 1 & 3\\ 7 & 1 & 9 & 0 & 7 & 8 & 9 & 5 & 9 & 9\\ 8 & 6 & 6 & 1 & 0 & 13 & 5 & 1 & 3 & 12\\ 1 & 4 & 7 & 3 & 2 & 0 & 5 & 2 & 5 & 2\\ 9 & 7 & 8 & 8 & 7 & 1 & 0 & 1 & 11 & 4\\ 12 & 11 & 3 & 3 & 7 & 1 & 6 & 0 & 3 & 8\\ 5 & 4 & 2 & 4 & 4 & 5 & 3 & 5 & 0 & 7\\ 2 & 7 & 5 & 11 & 8 & 7 & 9 & 8 & 4 & 0 \end{array}). \end{array}$$

The specific decoding steps are described as follows:

(1) Get the reachable matrices of AGVs

The travel path of AGV1 is 0 – −6 – −2 – −0, comparing the path with matrix D, value 1 represents the AGV1 through the station, otherwise represented with value 0. So the reachable matrix *X*_1_ of AGV1 can be obtained:

$$\begin{array}{c} X_{1}=(\begin{array}{cccccccccc} \infty & 0 & 0 & 0 & 0 & 0 & 1 & 0 & 0 & 0\\ 0 & 0 & 0 & 0 & 0 & 0 & 0 & 0 & 0 & 0\\ 1 & 0 & 0 & 0 & 0 & 0 & 0 & 0 & 0 & 0\\ 0 & 0 & 0 & 0 & 0 & 0 & 0 & 0 & 0 & 0\\ 0 & 0 & 0 & 0 & 0 & 0 & 0 & 0 & 0 & 0\\ 0 & 0 & 0 & 0 & 0 & 0 & 0 & 0 & 0 & 0\\ 0 & 0 & 1 & 0 & 0 & 0 & 0 & 0 & 0 & 0\\ 0 & 0 & 0 & 0 & 0 & 0 & 0 & 0 & 0 & 0\\ 0 & 0 & 0 & 0 & 0 & 0 & 0 & 0 & 0 & 0\\ 0 & 0 & 0 & 0 & 0 & 0 & 0 & 0 & 0 & 0 \end{array}). \end{array}$$

Similarly, we can get other AGVs reachable Matrices *X*_2_, *X*_3_, *X*_4_, *X*_5_.

(2) Get the path distance matrices of AGVs

Multiplying the matrix *X*_*k*_ (*k* = 1, 2, ⋯, 5) with *D* obtains the path distance matrix *D*_*k*_ (*k* = 1, 2, ⋯, 5) of each AGV.

For example, the path distance matrix for AGV1 is

$$\begin{array}{c} D_{1}=(\begin{array}{cccccccccc} \infty & 0 & 0 & 0 & 0 & 0 & 6 & 0 & 0 & 0\\ 0 & 0 & 0 & 0 & 0 & 0 & 0 & 0 & 0 & 0\\ 3 & 0 & 0 & 0 & 0 & 0 & 0 & 0 & 0 & 0\\ 0 & 0 & 0 & 0 & 0 & 0 & 0 & 0 & 0 & 0\\ 0 & 0 & 0 & 0 & 0 & 0 & 0 & 0 & 0 & 0\\ 0 & 0 & 0 & 0 & 0 & 0 & 0 & 0 & 0 & 0\\ 0 & 0 & 8 & 0 & 0 & 0 & 0 & 0 & 0 & 0\\ 0 & 0 & 0 & 0 & 0 & 0 & 0 & 0 & 0 & 0\\ 0 & 0 & 0 & 0 & 0 & 0 & 0 & 0 & 0 & 0\\ 0 & 0 & 0 & 0 & 0 & 0 & 0 & 0 & 0 & 0 \end{array}). \end{array}$$

(3) Compute the path distance of AGVs

The path distance of AGV1 is

$$\begin{array}{c} Z_{1}=6+3+8=17, \end{array}$$

similarly,

$$\begin{array}{c} Z_{2}=3+12=15, \end{array}$$

$$\begin{array}{c} Z_{3}=4+4+5=13, \end{array}$$

$$\begin{array}{c} Z_{4}=12+7+8=27, \end{array}$$

$$\begin{array}{c} Z_{5}=5+5+2=12, \end{array}$$

and the total path distance of all AGVs is

$$\begin{array}{c} Z=Z_{1}+Z_{2}+Z_{3}+Z_{4}+Z_{5}=84. \end{array}$$

## Simulation

In simulation, we set up the production scene with 5 AGVs and 50 workstations, and meet the requirements listed in the Section 2 and the double-path constraints. Set the population size is 200, applying the proposed new genetic algorithm to perform path optimization.

**Fig 7:**
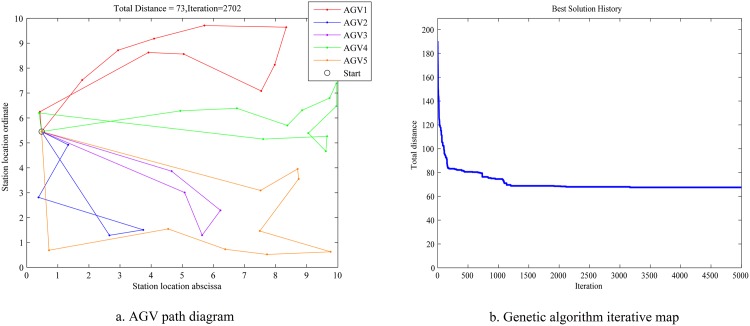
AGV diagram and map of the new genetic algorithm.

**Fig 8:**
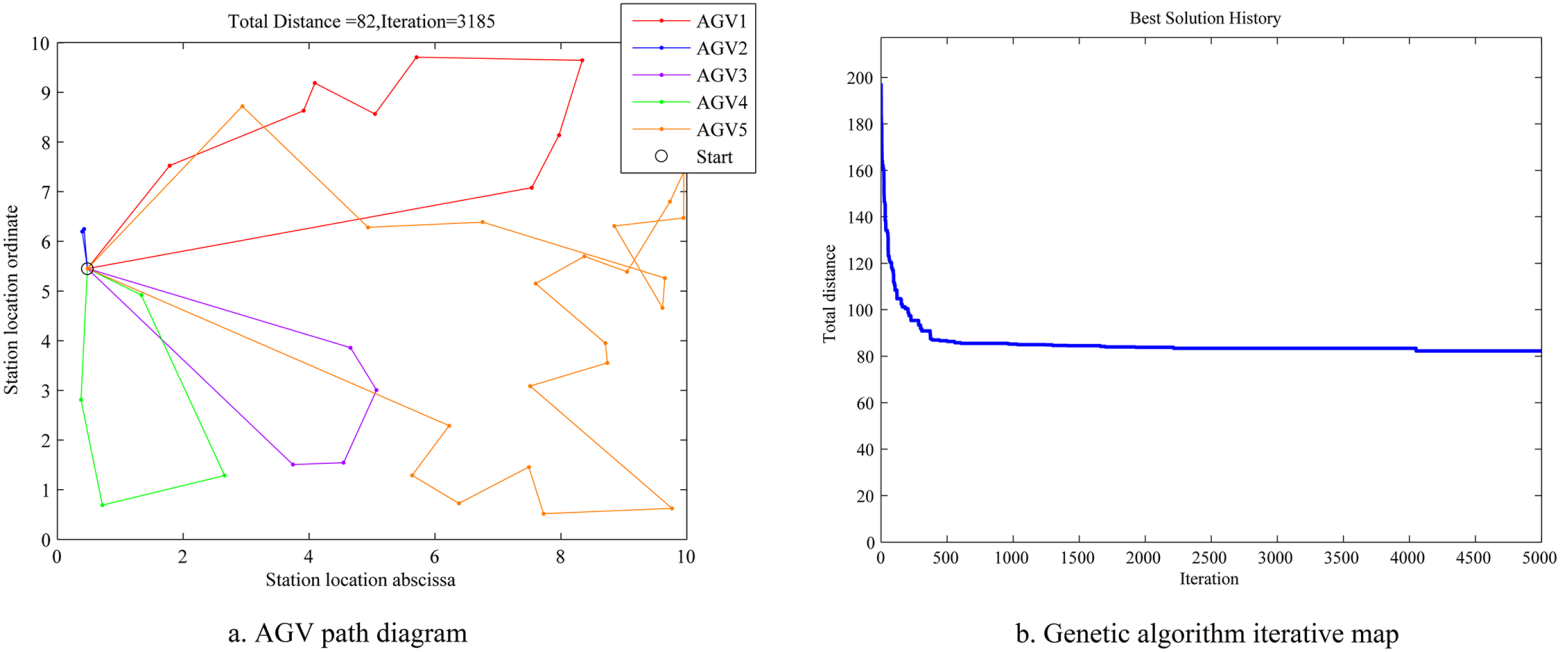
AGV diagram and map of the traditional genetic algorithm.

**Fig 9:**
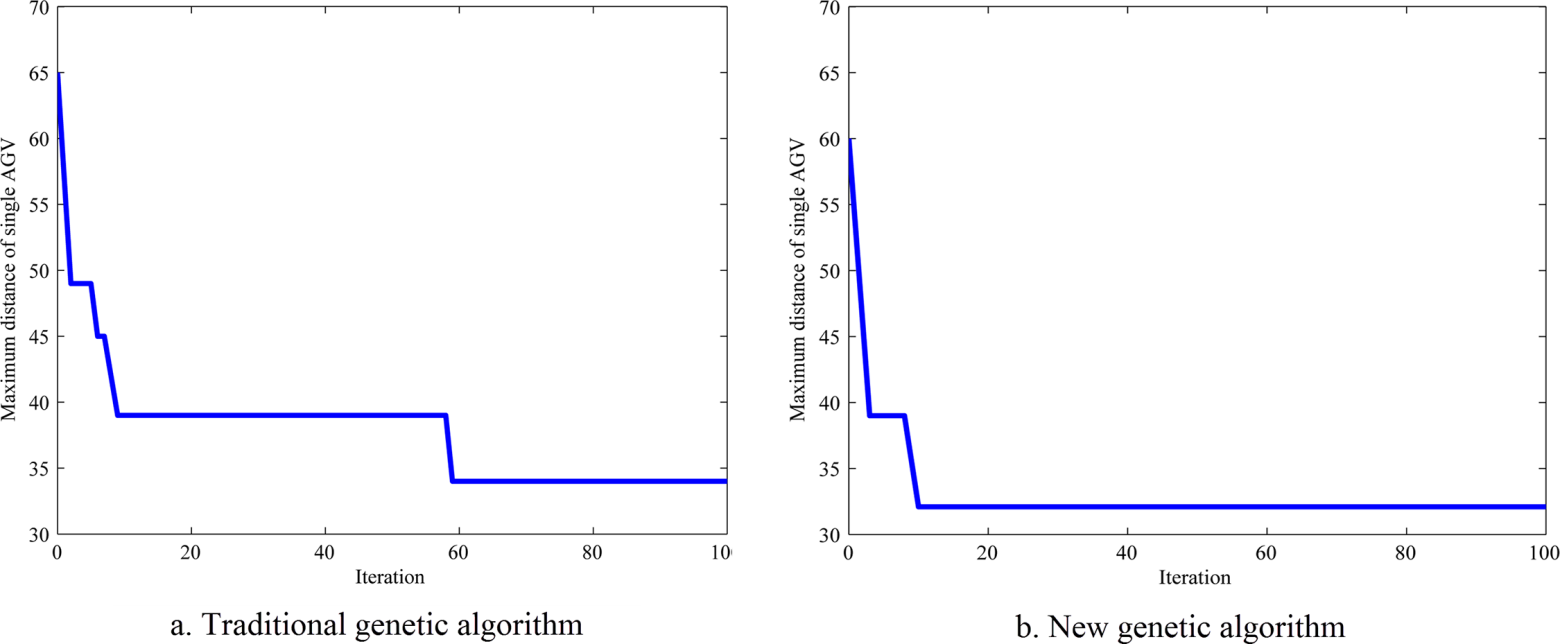
Maximum distance of single AGV.

**Fig 10:**
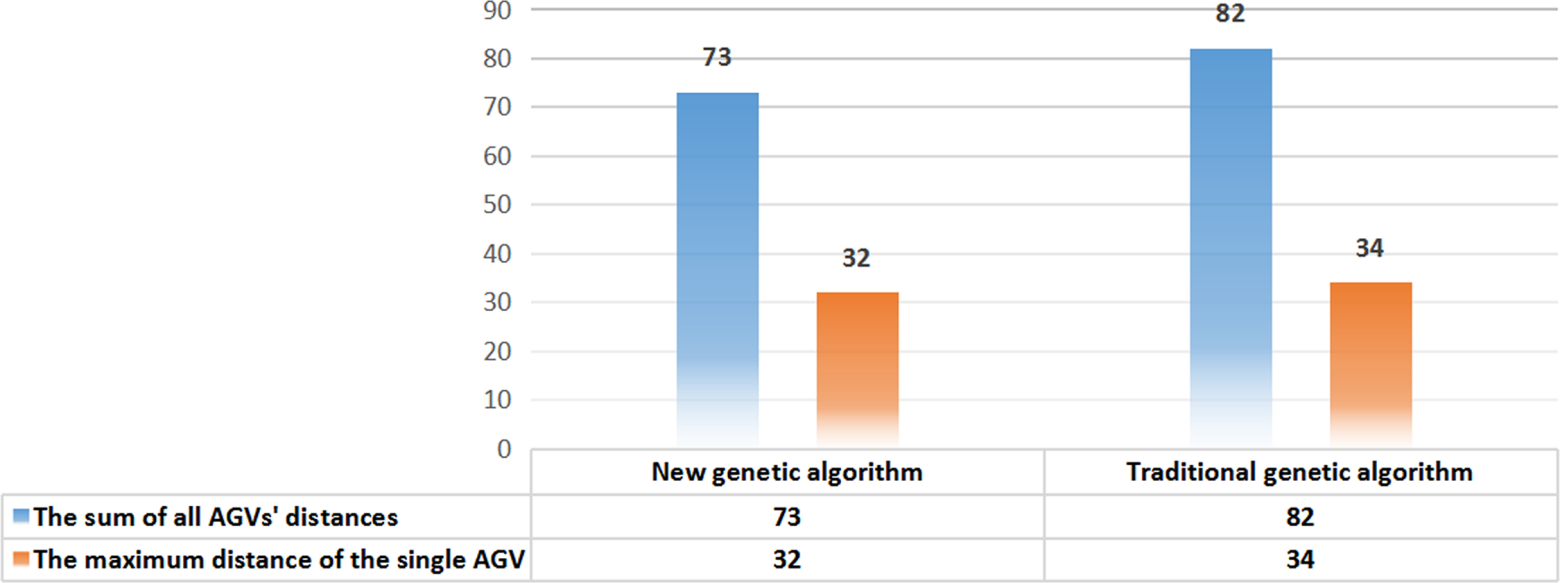
Distance comparison between two algorithms.

The simulation results for two genetic algorithms are drawn in Figs 7–10.

Figs 7 and 8 show that, at 3000 iterations, the total path distance is 72 for the new genetic algorithm, and is 86 for the traditional genetic algorithm.

Fig 9 shows that, at 60 iterations, the maximum distance of single AGV is 34 for the traditional genetic algorithm, and is 32 for the new genetic algorithm.

Fig 10 shows the distance comparison between the two algorithms using bar graphs.

From the simulation, we can get:

(1) The improved genetic algorithm have the shorter total path distance than that of the traditional genetic algorithm.

(2) The convergence speed of the improved genetic algorithm is faster than that of the traditional genetic algorithm.

## Conclusions

We recast the multi-AGV path planning problem into the framework of an genetic algorithm to investigate the improved genetic algorithm on multi-AGV path optimization. In the improved genetic algorithm, by using three-exchange crossover heuristic operators with more information than that of the traditional two-exchange crossover heuristic operators, we get more optimal offsprings. By exerting double-path constraints of both minimizing the total path distance of all AGVs and minimizing each AGV path distance, we gain an optimal shortest total path distance in AGV delivery task. The simulation results show that all AGV path distance and the longest single AGV path distance are shortened by using the improved genetic algorithm.

## Supporting information

S1 File Program of AGV diagram and map. (ZIP)

S2 File Program of maximum distance of single AGV. (ZIP)
